# Supplementary material for: Experimental glycopeptide antibiotic EVG7 prevents recurrent Clostridioides difficile infection by sparing members of the Lachnospiraceae family
Source: Nat Commun. 2025 Oct 10;16:9017. doi: 10.1038/s41467-025-64067-w (PMC12514160; doi:10.1038/s41467-025-64067-w)
Supplement: Supplementary file 1 — Supplementary Information [file 41467_2025_64067_MOESM1_ESM.pdf]

Supplementary Information

Experimental glycopeptide antibiotic EVG7 prevents recurrent *Clostridioides difficile* infection by sparing members of the *Lachnospiraceae* family

Contents

Supplementary Fig. 1: Low dose oral EVG7 outperforms high dose oral EVG7 in a mouse model of rCDI
Supplementary Table 1: In vitro antimicrobial activity against human *C. difficile* isolates
Supplementary Table 2: Clinical scoring chart for mice infected with *C. difficile*
Supplementary Table 3: In vitro antibacterial activity against human commensal gut bacteria
Supplementary Table 4: Reagents used in this work
Supplementary References

Suppl. Fig. 1: Low dose oral EVG7 outperforms high dose oral EVG7 in a mouse model of rCDI.

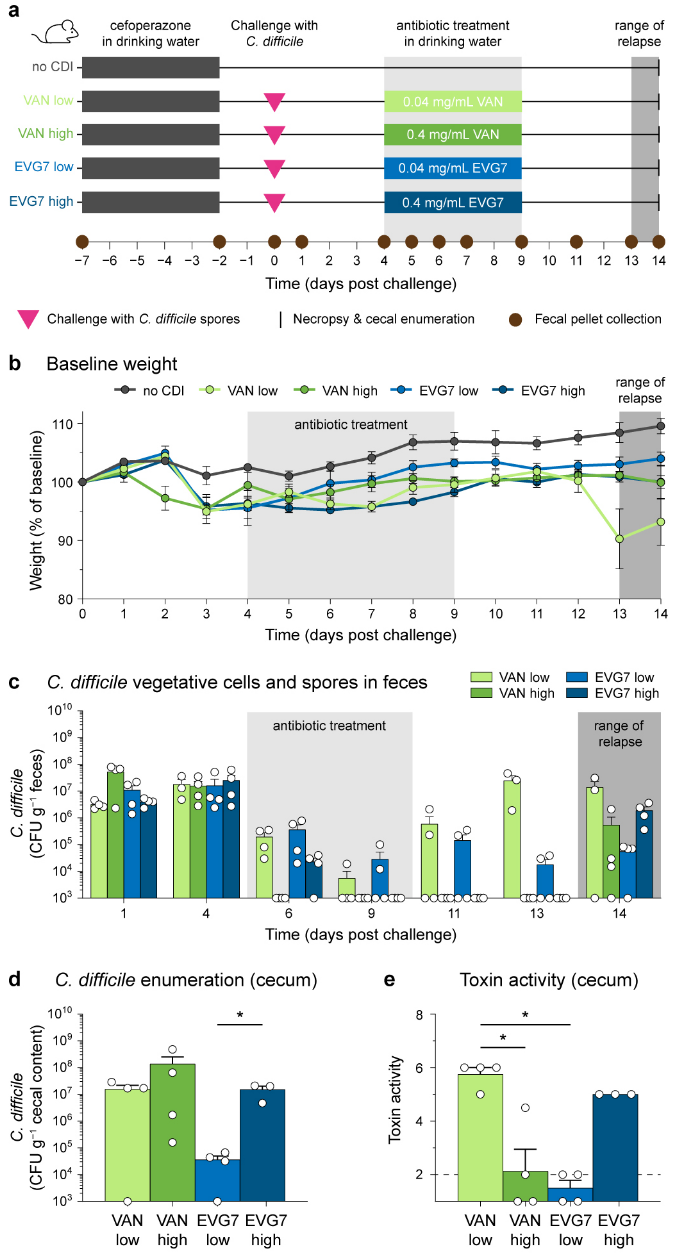

a, Timeline for mouse model of recurrent *C. difficile* infection (rCDI). Male C57BL/6J mice (n = 4 animals per group) were treated with cefoperazone in drinking water ad libitum for 5 days followed by a 2-day washout. All groups except the healthy ('no CDI', grey) group were challenged on day 0 with approximately 10^5 *C. difficile* spores (strain 630) via oral gavage. Treatment with 0.04 mg/mL vancomycin ('VAN low', light green), 0.4 mg/mL vancomycin ('VAN high', green), 0.04 mg/mL EVG7 ('EVG7 low', blue), or 0.4 mg/mL EVG7 ('EVG7 high', navy) was administered in the drinking water ad libitum from days 4–9. Mice were monitored for clinical signs of CDI for the duration of the study. All animals were euthanized and necropsied on day 14. **b**, Baseline weight loss of mice (n = 4 mice per group). Data are mean ± standard deviation. **c**, Total spores and vegetative cells of *C. difficile* in colony-forming units (CFUs) per gram of fecal content. Each dot (n = 4 per group) represents an individual fecal sample, bars represent the mean with error bars showing the standard deviation. Stool was not collected from deceased or very sick animals. Values below LOD were set to 1000. **d**, Total spores and vegetative cells of *C. difficile* in CFUs per gram of cecal content. Each dot represents an individual cecal sample (n = 4 animals per group), bars represent the mean with error bars showing the standard deviation. Values below LOD were set to 1000 for graphical and statistical purposes. Significance was determined by a one-sided Brown-Forsythe and Welch's ANOVA followed by a Dunnett T3 multiple posttest. **e**, Toxin activity in cecal content as determined by Vero cell cytotoxicity assay. Visual presentation as described for panel (**d**). Significance was determined by a one-sided Kruskal-Wallis ANOVA followed by a Dunn's multiple comparisons posttest. Error bars represent the standard deviation from the mean. The dashed line represents the limit of detection for the toxin activity (reciprocal log titer). For all statistical tests: $*P \leq 0.05$. Graphs were generated in GraphPad Prism 10 and further compiled in Adobe Illustrator 2024. Source data are provided as a Source Data file.

Supplementary Table 1: In vitro antimicrobial activity against human *C. difficile* isolates.

Strain ID	Phylogenic clade	PCR ribotype	MIC VAN (mg/L)	MIC EVG7 (mg/L)
LL-001	1	001	1	0.125
LL-002	1	002	1	0.125
LL-005	1	005	1	0.125
LL-010	1	010	1	0.125
LL-012	1	012	1	0.125
LL-014	1	014	1	0.125
LL-015	1	015	2	0.25
LL-020	1	020	1	0.125
LL-081	1	081	1	0.063
LL-106	1	106	1	0.125
LL-016	2	016	0.5	0.125
LL-019	2	019	1	0.125
LL-027	2	027	1	0.125
LL-036	2	036	1	0.063
CL-176	2	176	1	0.125
Greece-19-181	2	181	0.5	0.063
LL-198	2	198	1	0.125
L-ref-244	2	244	1	0.125
LL-023	3	023	0.5	0.063
LL-058	3	058	1	0.125
LL-063	3	063	1	0.125
LL-017	4	017	1	0.063
LL-060	4	060	1	0.063
LL-088	4	088	0.5	0.063
LUMCMM25 0031	4	130	1	0.125
LL-033	5	033	1	0.125
LL-045	5	045	1	0.125
LL-066	5	066	1	0.125
LL-078	5	078	1	0.125
LL-126	5	126	1	0.125
LUMCMM25 0032	5	288	1	0.125
LUMCMM16 0013[1]	C-I	N/A	1	0.063
LUMCMM21 0001[1]	C-II	151	1	0.125
LUMCMM19 2333[1]	C-II	151	1	0.125
ATCC 70057 (control)	1*	038	1	0.125

Minimal Inhibitory Concentration (MIC) of vancomycin (VAN) and EVG7 against *C. difficile* clinical isolates, determined by agar dilution method in Brucella Blood Agar (BBA) supplemented with sheep blood, hemin, and vitamin K. The isolates tested belong to a well-established reference collection[2,3] supplemented with clinical isolates collected by the National Expertise Center for *C. difficile* infections (hosted at the LUMC, Leiden, NL)[1]. N/A = not available. *Clade inferred from ATCC-listed PCR ribotype[4]; MLST or clade is not listed on ATCC website (https://www.atcc.org/products/700057). Data accompanying Fig. 1.

Supplementary Table 2: Clinical scoring chart for mice infected with *C. difficile*.

Category	Parameter score*			
	0	1	2	3
Weight loss**	<1.5%	1.5–2.5%	2.5–5%	>5%
Activity	Bright, alert, reactive	Alert, but slow moving	Lethargic, but moves readily when stressed	Lethargic and shaky, hesitant to move when stressed
Posture	Normal	Back slanted	Hunched	Hunched with nose down
Coat	Normal/well groomed	Piloerection	Rough coat	Very ruffled and ungroomed
Diarrhea	Normal	Soft stool or change in color (yellow)	Wet tail/mucous or blood present in stool	Liquid or no stool present

*Clinical score = sum of all parameter scores. Maximum clinical score is 15. Mice with clinical score ≥ 12 should be considered for euthanasia. **Weight loss percentage is calculated relative to previous day (not relative to day 0). Data accompanying Fig. 2C.

Supplementary Table 3: In vitro antibacterial activity against human commensal gut bacteria.

	Phylum	Family	ID	Species	MIC (mg/L) VAN	MIC (mg/L) EVG7	MIC (fold-change)
Gram-positive bacteria	Firmicutes	*Peptostreptococcaceae*	IS029	*Clostridioides difficile* ATCC 70057	1	0.125	8
			IS030	*Clostridioides difficile* NCTC 15085	1	0.25	4
		Clostridiaceae	IS016	*Clostridium hylemonae* DSM15053	1	0.125	8
			IS017	*Clostridium ramosum*	8	1	8
			IS018	*Clostridium scindens* DSM5676	2	0.5	4
		Eubacteriaceae	IS020	*Eubacterium hallii*	2	0.5	4
		Oscillospiraceae	IS021	*Flavonifractor plautii*	8	2	4
		Lachnospiraceae	IS019	*Coprococcus eutactus*	2	0.5	4
			IS026	Unclassified *Lachnospiraceae sp.* 1	0.25	0.125	2
			IS027	Unclassified *Lachnospiraceae sp.* 2	0.5	0.125	4
			IS028	Unclassified *Lachnospiraceae sp.* 3	0.5	0.25	2
		Lactobacillaceae	IS022	*Lactobacillus gasseri*	2	0.5	4
		Streptococcaceae	IS023	*Lactococcus garviae*	1	0.25	4
		Staphylococcaceae	IS025	*Staphylococcus aureus* NCTC 12973	2	1	2
	Actinobacteria	*Bifidobacteriaceae*	IS004	*Bifidobacterium sp.*	1	0.5	2
			IS012	*Bifidobacterium longum*	0.5	0.125	4
			IS013	*Bifidobacterium longum infantis*	0.5	0.125	4
			IS014	*Bifidobacterium adolescentis*	1	1	1
		Coriobacteriaceae	IS015	*Collinsella aerofaciens*	0.5	0.063	8
Gram-negative bacteria	Pseudomonadota	*Enterobacteriaceae*	IS031	*Escherichia coli* NCTC 12241	>32	>16	–
	Bacteroidota	*Bacteroidaceae*	IS005	*Bacteroides finegoldii*	>32	>16	–
			IS006	*Bacteroides dorei*	32	>16	–
			IS007	*Bacteroides fragilis* ATCC 25285	32	>16	–
			IS008	*Bacteroides ovatus*	>32	>16	–
			IS009	*Bacteroides thetaiotaomicron*	>32	>16	–
			IS010	*Bacteroides uniformis*	>32	>16	–
			IS011	*Bacteroides vulgatus*	32	>16	–
		Rikenellaceae	IS003	*Alistipes shahii*	>32	>16	–
			IS002	*Alistipes onderdonkii*	>32	8	>4
		Prevotellaceae	IS024	*Paraprevotella clara*	>32	0.25	>128
	Verrucomicrobiota	*Akkermansiaceae*	IS001	*Akkermansia muciniphila*	32	0.25	128

Minimal Inhibitory Concentration (MIC) of vancomycin (VAN) and EVG7 against commensal clinical isolates determined by agar dilution method on Fastidious Anaerobe Agar with horse blood (FAA-HB)[5]. All isolates in this panel have been obtained from public repositories (catalog number listed) or isolated from human donors at the Leiden University Medical Center (LUMC) in the Netherlands. Data accompanying Fig. 4.

Supplementary Table 4: Reagents used in this work.

	Reagent	Supplier	Catalogue number
	EVG7	in-house synthesis[6]	
MIC assays	Vancomycin Hydrochloride	VWR	Applichem A1839
	Tryptic Soy Sheep Blood Agar plates (TSS)	bioMérieux	43009
	Schaedlers anaerobic broth	Oxoid/ThermoFisher	CM0497B
	Saline 0.9%	In-house; prepared with Sodium chloride, Supelco	1.06404.1000
	Brucella Agar base	Oxoid/Thermofisher	CM0169
	Fastidious Anaerobe Agar base (FAA)	Biotrading Benelux BV	Neogen NCM0014a
	Sheep blood, defibrinated	Xebios Diagnostics GmbH	10000100/10000250
	Horse Blood, defibrinated	Xebios Diagnostics GmbH	2000100
	Hemin	SigmaAldrich	51280
	Vitamin K1	Carl Roth GmbH & Co	3804.2
Murine model of CDI (in vivo studies & analysis)	Vancomycin HCl	SigmaAldrich	V2002
	Clospore medium	in-house preparation[7]	
	Taurocholate BHI (TBHI)	in-house preparation[8]	
	Taurocholate, Cefoxitin, Cycloserine, and Fructose Agar (TCCFA)	in-house preparation[8]	
	Brain Heart Infusion (BHI)	BD Life Sciences	241810
	Cefoperazone	MP Biomedicals	02199695-CF
	Drinking water	Gibco Laboratories	15230
	phosphate-buffered saline (PBS)	ThermoFisher	10-010-049
	DMEM media	Gibco laboratories	11965-092
	Fetal bovine serum (FBS)	Gibco Laboratories	16140-071
	1% Penicillin-Streptomycin	Gibco Laboratories	15070-063
	0.25% Trypsin-EDTA	Gibco Laboratories	25200-056
	C. difficile Antitoxin Kit	TechLabs	T5000
	C. difficile Toxin A	List Biological Labs	152C
	Bacto Agar	Becton Dickinson	214010
	Bacto Proteose Peptone	Becton Dickinson	211684
	Cefoxitine	SigmaAldrich	C47856
	D-Cycloserine	SigmaAldrich	C6880
	Fructose	Fisher	L95500
	KH_2PO_4	Fisher	P285-500
	$MgSO_4$ (anhydrous)	SigmaAldrich	M2643
	Na_2HPO_4	SigmaAldrich	S-0876
	NaCl	Fisher	S640-3
	Taurocholate	SigmaAldrich	T4009

Supplementary References

1. Ducarmon, Q. R., van der Bruggen, T., Harmanus, C., Sanders, I. M. J. G., Daenen, L. G. M., Fluit, A. C., Vossen, R. H. A. M., Kloet, S. L., Kuijper, E. J. & Smits, W. K. *Clostridioides difficile* infection with isolates of cryptic clade C-II: a genomic analysis of polymerase chain reaction ribotype 151. *Clinical Microbiology and Infection* **29**, 538.e531-538.e536 (2023).

2. Knetsch, C. W., Terveer, E. M., Lauber, C., Gorbalenya, A. E., Harmanus, C., Kuijper, E. J., Corver, J. & van Leeuwen, H. C. Comparative analysis of an expanded *Clostridium difficile* reference strain collection reveals genetic diversity and evolution through six lineages. *Infection, Genetics and Evolution* **12**, 1577–1585 (2012).

3. Baktash, A., Corver, J., Harmanus, C., Smits, W. K., Fawley, W., Wilcox, M. H., Kumar, N., Eyre, D. W., Indra, A., Mellmann, A. & Kuijper, E. J. Comparison of Whole-Genome Sequence-Based Methods and PCR Ribotyping for Subtyping of *Clostridioides difficile*. *Journal of Clinical Microbiology* **60**, e01737-01721 (2022).

4. Putsathit, P., Neela, V. K., Joseph, N. M. S., Ooi, P. T., Ngamwongsatit, B., Knight, D. R. & Riley, T. V. Molecular epidemiology of *Clostridium difficile* isolated from piglets. *Veterinary Microbiology* **237**, 108408 (2019).

5. Freeman, J., Sanders, I. M. J. G., Harmanus, C., Clark, E. V., Berry, A. M. & Smits, W. K. Antimicrobial susceptibility testing of *Clostridioides difficile*: a dual-site study of three different media and three therapeutic antimicrobials. *Clinical Microbiology and Infection* **6**, 1011–1017 (2025).

6. van Groesen, E., Mons, E., Kotsogianni, I., Arts, M., Tehrani, K. H. M. E., Wade, N., Lysenko, V., Stel, F. M., Zwerus, J. T., De Benedetti, S., Bakker, A., Chakraborty, P., van der Stelt, M., Scheffers, D.-J., Gooskens, J., Smits, W. K., Holden, K., Gilmour, P. S., Willemse, J., Hitchcock, C. A., van Hasselt, J. G. C., Schneider, T. & Martin, N. I. Semisynthetic guanidino lipoglycopeptides with potent in vitro and in vivo antibacterial activity. *Science Translational Medicine* **16**, eabo4736 (2024).

7. Perez, J., Springthorpe, V. S. & Sattar, S. A. Clospore: A Liquid Medium for Producing High Titers of Semi-purified Spores of *Clostridium difficile*. *Journal of AOAC International* **94**, 618–626 (2011).

8. Winston, J. A., Thanissery, R., Montgomery, S. A. & Theriot, C. M. Cefoperazone-treated Mouse Model of Clinically-relevant *Clostridium difficile* Strain R20291. *Journal of Visualized Experiments* **118**, e54850 (2016).
