# Supplementary material for: Experimental glycopeptide antibiotic EVG7 prevents recurrent Clostridioides difficile infection by sparing members of the Lachnospiraceae family
Source: Nat Commun. 2025 Oct 10;16:9017. doi: 10.1038/s41467-025-64067-w (PMC12514160; doi:10.1038/s41467-025-64067-w)
Supplement: Supplementary file 2 — Reporting Summary [file 41467_2025_64067_MOESM2_ESM.pdf]

nature portfolio

Corresponding author(s): Nathaniel Martin

Last updated by author(s): Aug 22, 2025

Reporting Summary

Nature Portfolio wishes to improve the reproducibility of the work that we publish. This form provides structure for consistency and transparency in reporting. For further information on Nature Portfolio policies, see our Editorial Policies and the Editorial Policy Checklist.

Statistics

For all statistical analyses, confirm that the following items are present in the figure legend, table legend, main text, or Methods section.

n/a	Confirmed	
☐	☒	The exact sample size (*n*) for each experimental group/condition, given as a discrete number and unit of measurement
☒	☐	A statement on whether measurements were taken from distinct samples or whether the same sample was measured repeatedly
☐	☒	The statistical test(s) used AND whether they are one- or two-sided<br>*Only common tests should be described solely by name; describe more complex techniques in the Methods section.*
☒	☐	A description of all covariates tested
☐	☒	A description of any assumptions or corrections, such as tests of normality and adjustment for multiple comparisons
☐	☒	A full description of the statistical parameters including central tendency (e.g. means) or other basic estimates (e.g. regression coefficient) AND variation (e.g. standard deviation) or associated estimates of uncertainty (e.g. confidence intervals)
☐	☒	For null hypothesis testing, the test statistic (e.g. *F*, *t*, *r*) with confidence intervals, effect sizes, degrees of freedom and *P* value noted<br>*Give P values as exact values whenever suitable.*
☒	☐	For Bayesian analysis, information on the choice of priors and Markov chain Monte Carlo settings
☒	☐	For hierarchical and complex designs, identification of the appropriate level for tests and full reporting of outcomes
☒	☐	Estimates of effect sizes (e.g. Cohen's *d*, Pearson's *r*), indicating how they were calculated

Our web collection on statistics for biologists contains articles on many of the points above.

Software and code

Policy information about availability of computer code

Data collection	No software was used for data collection
Data analysis	Statistical analyses of in vivo study data (Fig. 2 and Supplementary Fig. 1) were performed using GraphPad Prism 10 v.10.3.1.<br><br>Taxonomic profiling of microbiome data (Fig.3) was performed using "trimmomatic" v0.36 and "QIIME 2" v2024.10 with DADA2 and feature-classifier plugins. Statistical analyses of microbiome data were performed using R v4.1.2 and associated packages including "phyloseq" v1.38.0; "microbiome" v1.16.0; "microViz" v0.9.3.; "vegan" v2.6-4; ggvegan" v0.1.999; and "ggplot2" v3.3.6.

For manuscripts utilizing custom algorithms or software that are central to the research but not yet described in published literature, software must be made available to editors and reviewers. We strongly encourage code deposition in a community repository (e.g. GitHub). See the Nature Portfolio guidelines for submitting code & software for further information.

Data

Policy information about availability of data

All manuscripts must include a data availability statement. This statement should provide the following information, where applicable:

- Accession codes, unique identifiers, or web links for publicly available datasets
- A description of any restrictions on data availability
- For clinical datasets or third party data, please ensure that the statement adheres to our policy

The demultiplexed 16S rRNA gene amplicon sequencing data are available from the European Nucleotide Archive under accession number PRJEB86983.

Research involving human participants, their data, or biological material

Policy information about studies with human participants or human data. See also policy information about sex, gender (identity/presentation), and sexual orientation and race, ethnicity and racism.

Reporting on sex and gender	N.A.
Reporting on race, ethnicity, or other socially relevant groupings	N.A.
Population characteristics	N.A.
Recruitment	N.A.
Ethics oversight	In this study, bacteria isolated from human stool were used (not human stool itself). These bacteria were originally isolated from stool obtained from the Netherlands Donor Feces Bank (https://www.ndfb.nl/), independent from the present study. Written informed consent was obtained for using donor fecal samples from the Netherlands Donor Feces Bank (https://www.ndfb.nl/) for research purposes, and approved by the Medical Ethics Committee at Leiden University Medical Center (P15.145).

Note that full information on the approval of the study protocol must also be provided in the manuscript.

Field-specific reporting

Please select the one below that is the best fit for your research. If you are not sure, read the appropriate sections before making your selection.

☒ Life sciences ☐ Behavioural & social sciences ☐ Ecological, evolutionary & environmental sciences

For a reference copy of the document with all sections, see nature.com/documents/nr-reporting-summary-flat.pdf

Life sciences study design

All studies must disclose on these points even when the disclosure is negative.

Sample size	Sample sizes in the in vivo studies were selected based on published results in the field and preliminary experimentation. No samples size calculation was performed.<br>In vivo C. difficile CFUs were compared from n=4-12 mice depending on the treatment. The number of mice used in this study was n=4-12 per group and was necessary for statistical power and to control for cage-to cage variation. See methods and Fig. 2 and Fig. S1 legend for more details.
Data exclusions	Stool collection was not possible for all animals on some days, because sick animals often do not defecate: details and exceptions are provided in the 'Source Data document'.<br><br>Animals that expired before the end of the study were not necropsied and their weight/clinical score/stool was not collected after death:<br>In Fig. 2, mouse 431-3 (VAN group) met clinical endpoint on day 14 and not necropsied; mouse 431-4 (VAN group) died from non-CDI related causes on day 4 and not necropsied.<br>In Supplementary Fig. 1, mouse 384-3 (no CDI group) had hydrocephalus and was euthanized accordingly before the end of the cef week (day -6); mouse 383-3 (VAN low group) was necropsied on day 13 due to reaching humane endpoint; mouse 386-2 (EVG7 high group) met endpoint before necropsy and was not necropsied.<br><br>Stool samples from day 18 of the EVG7-treated mice were excluded from the principal response curve (Fig.3F) since the necropsy day of the reference group (vancomycin-treated mice) was on day 14/15.
Replication	Data points in figures from all biological replicates are displayed for data in Fig. 2. Replication of the in vivo mouse experiment in Fig. 2 was performed successfully.
Randomization	Mice were assigned a treatment group at random upon arrival. All mice were selected for necropsy from different cages post C. difficile challenge to account for cage to cage variation.

Blinding	We were not blinded to the samples in the in vivo studies when running all experiments. We were not blinded to the groups of mice in this study to prevent cross contamination. Additionally, C. difficile infected mice exhibit clinical signs which are quite obvious (lethargy, hunched posture, ruffled fur, wet fecal pellets decorating the cage walls, conjunctivitis, etc.). We were blinded to all downstream analysis including bacterial enumeration measurements and toxin activity of mouse cecal content seen in Fig. 2D-F and Fig. S1C-E.

Reporting for specific materials, systems and methods

We require information from authors about some types of materials, experimental systems and methods used in many studies. Here, indicate whether each material, system or method listed is relevant to your study. If you are not sure if a list item applies to your research, read the appropriate section before selecting a response.

Materials & experimental systems

n/a	Involved in the study	
☒	☐	Antibodies
☒	☐	Eukaryotic cell lines
☒	☐	Palaeontology and archaeology
☐	☒	Animals and other organisms
☒	☐	Clinical data
☒	☐	Dual use research of concern
☒	☐	Plants

Methods

n/a	Involved in the study	
☒	☐	ChIP-seq
☒	☐	Flow cytometry
☒	☐	MRI-based neuroimaging

Animals and other research organisms

Policy information about studies involving animals; ARRIVE guidelines recommended for reporting animal research, and Sex and Gender in Research

Laboratory animals	C57BL/6J mice, male, 4–5 weeks old, were purchased from Jackson Laboratories. Male mice were housed in a room with an average temperature of 70F and 35% humidity.
Wild animals	Study did not involve wild animals
Reporting on sex	The data generated in this study was using only male animals. The findings reported in our manuscript are, however, expected to apply equally to all sexes/genders. We have used both male and female mice in our experiments over the past decade and have not seen any differences in disease outcome. To adhere to rigorous statistical analysis and to reduce the number of animals used in research per the 3Rs (Replacement, Reduction, and Refinement), we chose to only use male mice in this study. We have also clearly detailed this in the methods section.
Field-collected samples	N.A.
Ethics oversight	All animal experiments were conducted in the Laboratory Animal Facilities located on the North Carolina State University (NCSU) College of Veterinary Medicine (CVM) campus (Raleigh, USA). The animal facilities are equipped with a full-time animal care staff coordinated by the Laboratory Animal Resources (LAR) division at NCSU. The NCSU CVM is accredited by the Association for the Assessment and Accreditation of Laboratory Animal Care International (AAALAC). Trained animal handlers in the facility fed and assessed the status of animals several times per day. Those assessed as moribund were humanely euthanized by CO_2 asphyxiation. This protocol is approved by NC State's Institutional Animal Care and Use Committee (IACUC).

Note that full information on the approval of the study protocol must also be provided in the manuscript.

Plants

Seed stocks	N.A.
Novel plant genotypes	N.A.
Authentication	N.A.
